# Exercise as a Molecular Therapeutic Tool in MASLD: From Signaling Pathways to Clinical Translation—A Narrative Review

**DOI:** 10.3390/biomedicines14030577

**Published:** 2026-03-04

**Authors:** Héctor Fuentes-Barría, Raúl Aguilera-Eguía, Cherie Flores-Fernández, Lissé Angarita-Davila, Miguel Alarcón-Rivera

**Affiliations:** 1 Centro de Investigación en Medicina de Altura (CEIMA), Universidad Arturo Prat, Iquique 1110939, Chile; 2 Departamento de Salud Pública, Facultad de Medicina, Universidad Católica de la Santísima Concepcion, Concepcion 3349001, Chile; raguilerae@ucsc.cl; 3 Departamento Gestión de la Información, Universidad Tecnológica Metropolitana, Santiago 7550000, Chile; cflores@utem.cl; 4 Escuela de Nutrición y Dietética, Facultad de Medicina, Universidad Andres Bello, Concepcion 3349001, Chile; lisse.angarita@unab.cl; 5 Escuela de Ciencias del Deporte y Actividad Física, Facultad de Salud, Universidad Santo Tomas, Talca 3460000, Chile; mrivera3@santotomas.cl

**Keywords:** exercise, non-alcoholic fatty liver disease, mitochondria, liver, insulin resistance, inflammation

## Abstract

Physical exercise is a potent non-pharmacological strategy for the prevention and management of Metabolic dysfunction—associated steatotic liver disease (MASLD), a multifactorial disorder characterized by hepatic lipid accumulation, insulin resistance, oxidative stress, and chronic inflammation. Emerging evidence demonstrates that the benefits of exercise extend beyond caloric expenditure and are largely mediated by coordinated molecular and cellular adaptations within the liver and peripheral tissues. This review synthesizes current knowledge on the mechanisms through which exercise modulates MASLD pathophysiology, emphasizing intracellular signaling pathways, mitochondrial remodeling, antioxidant defenses, and myokine-driven muscle–liver crosstalk. Exercise induces acute and chronic activation of pathways such as AMPK, PGC-1α, Nrf2, and Akt, resulting in enhanced mitochondrial biogenesis, improved fatty acid oxidation, restored insulin signaling, and reduced inflammatory and oxidative stress. Repeated skeletal muscle contraction stimulates the release of myokines—including irisin, IL-6, and FGF21—that act through endocrine and paracrine routes to regulate hepatic lipid metabolism, promote systemic metabolic flexibility, and attenuate disease progression. Epigenetic modifications and exercise-responsive microRNAs further contribute to long-term hepatic metabolic reprogramming. Collectively, these molecular adaptations position exercise as a systemic, disease-modifying stimulus capable of restoring hepatic homeostasis, slowing the transition from steatosis to NASH and fibrosis, and improving long-term metabolic health. Understanding these mechanisms provides a foundation for developing targeted, personalized exercise-based interventions in the clinical management of MASLD.

## 1. Introduction

Non-alcoholic fatty liver disease (NAFLD) has emerged as the most prevalent chronic liver disorder worldwide, encompassing a broad pathological spectrum ranging from simple steatosis to non-alcoholic steatohepatitis (NASH), progressive fibrosis, cirrhosis, and hepatocellular carcinoma [1,2]. Closely associated with obesity, insulin resistance, and metabolic syndrome, NAFLD is now recognized as Metabolic Dysfunction–Associated Steatotic Liver Disease (MASLD), a condition whose complications pose a substantial clinical and socioeconomic burden and whose global incidence continues to rise [3,4,5]. Despite substantial advances in understanding its pathogenesis, effective pharmacological therapies remain limited, underscoring the urgent need for alternative and complementary therapeutic strategies targeting the underlying molecular drivers of disease progression [6].

At the molecular level, MASLD is characterized by a complex interplay between hepatic lipid overload, impaired insulin signaling, mitochondrial dysfunction, oxidative stress, and chronic low-grade inflammation [7,8,9]. Excessive influx of free fatty acids and increased de novo lipogenesis promote hepatocellular lipotoxicity, leading to endoplasmic reticulum stress, activation of inflammatory pathways such as nuclear factor kappa B (NF-κB), and enhanced production of pro-inflammatory cytokines [9,10,11]. Concurrently, disruption of insulin receptor substrate (IRS)—Protein Kinase B (Akt) signaling exacerbates hepatic insulin resistance, further amplifying lipid accumulation and gluconeogenesis [7]. Mitochondrial dysfunction and reduced oxidative capacity contribute to reactive oxygen species generation, perpetuating cellular injury and facilitating the transition from benign steatosis to NASH and fibrotic remodeling [8,12].

In recent years, physical exercise has gained recognition as a potent non-pharmacological intervention capable of modulating multiple pathogenic pathways involved in MASLD [13,14]. Beyond its well-established effects on body composition and energy balance, exercise exerts profound molecular actions on hepatic and extrahepatic tissues [15,16]. Accumulating evidence indicates that regular physical activity activates AMP-activated protein kinase (AMPK) signaling, suppresses sterol regulatory element-binding protein 1c (SREBP-1c)-mediated lipogenesis, enhances carnitine palmitoyltransferase-1 (CPT-1)-dependent fatty acid oxidation, and promotes mitochondrial biogenesis through peroxisome proliferator-activated receptor gamma coactivator-1 alpha (PGC-1α) [17,18,19]. These adaptations collectively improve hepatic lipid handling and metabolic flexibility

Importantly, exercise also attenuates inflammatory and oxidative pathways implicated in MASLD progression [20,21]. Reductions in tumor necrosis factor-alpha (TNF-α) and other pro-inflammatory mediators, alongside activation of nuclear factor erythroid 2-related factor 2 (Nrf2)-dependent antioxidant defenses, have been reported following structured exercise interventions [17,18,19]. Moreover, skeletal muscle acts as an endocrine organ during physical activity, releasing myokines such as irisin, interleukin-6 (IL-6), and fibroblast growth factor 21 (FGF21), which mediate crosstalk between muscle and liver and contribute to systemic metabolic improvements [22,23,24,25,26]. This emerging muscle–liver axis highlights exercise as a biologically active stimulus capable of reprogramming hepatic metabolism at the molecular level [24,25,26].

Despite growing interest in exercise-based interventions for MASLD, the precise molecular mechanisms underlying its hepatoprotective effects remain incompletely understood, and their translation into optimized clinical strategies is still evolving [13,14,24]. A comprehensive narrative synthesis integrating molecular signaling pathways with translational and therapeutic perspectives is therefore warranted.

Accordingly, this narrative review aims to summarize current evidence regarding the molecular adaptations induced by physical exercise in MASLD and to discuss their clinical and therapeutic implications. By integrating findings from experimental and human studies, we seek to provide a mechanistic framework supporting exercise as a disease-modifying intervention and to highlight future directions toward precision exercise medicine in the management of MASLD.

## 2. Pathophysiological Background of MASLD

**Figure 1:**
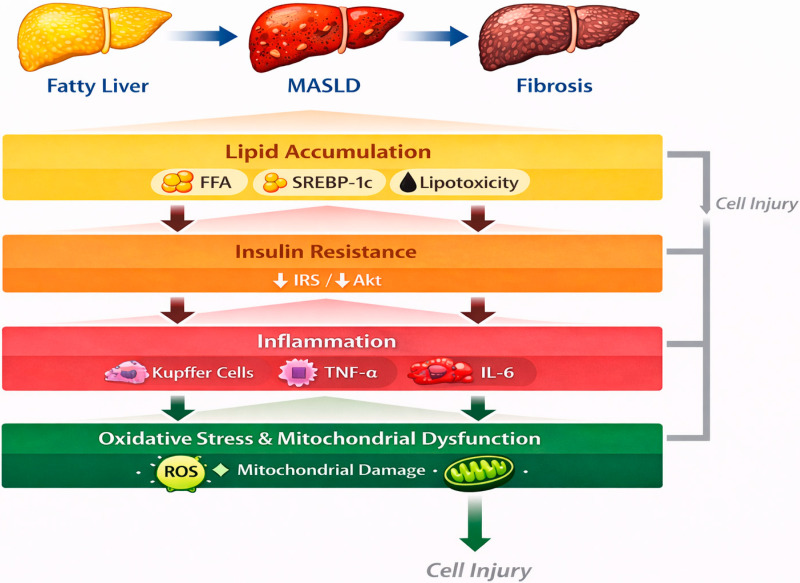
Pathophysiological Background of MASLD. Source: own elaboration. MASLD: Metabolic dysfunction—associated steatotic liver disease, FFA: Free Fatty Acids, SREBP-1c: suppresses sterol regulatory element-binding protein 1c, IRS: insulin receptor substrate, Akt: Protein Kinase B, TNF-α; Tumor Necrosis Factor alpha, IL-6: Interleukin-6, ROS: Reactive Oxygen Species.

MASLD is a multifactorial disorder arising from the convergence of metabolic, inflammatory, and oxidative disturbances within the liver, reflecting a profound disruption of hepatic homeostasis in the context of systemic metabolic dysfunction [27]. Its progression is driven by the dynamic interaction of excessive lipid accumulation, insulin resistance, chronic low-grade inflammation, and oxidative stress, which together create a self-perpetuating cycle of cellular injury and maladaptive repair [28]. These interrelated processes promote hepatocellular lipotoxicity, impair intracellular signaling networks, and activate stress-responsive pathways, ultimately facilitating the transition from simple steatosis to NASH, progressive fibrosis, and advanced liver disease [2,10,29]. Rather than following a strictly linear trajectory, MASLD develops through multiple parallel and interconnected mechanisms acting on a metabolically vulnerable hepatic environment, consistent with the “multiple-hit” model of disease pathogenesis. In this framework, genetic susceptibility, altered adipose tissue signaling, gut-derived factors, and mitochondrial dysfunction further modulate disease severity and progression [9,30]. The resulting hepatic microenvironment is characterized by impaired metabolic flexibility, heightened inflammatory tone, and reduced antioxidant capacity, which collectively accelerate structural and functional deterioration of the liver [31]. Understanding MASLD as a systems-level disorder driven by overlapping molecular insults provides a critical conceptual basis for therapeutic strategies aimed at simultaneously targeting metabolic, inflammatory, and oxidative pathways [32,33,34] (Figure 1).

### 2.1. Hepatic Lipid Accumulation

Excessive hepatic lipid accumulation represents the initiating hallmark of MASLD and reflects a profound imbalance between fatty acid influx, de novo lipogenesis, oxidation, and lipid export [35,36,37]. Under conditions of systemic metabolic dysfunction, increased delivery of free fatty acids from insulin-resistant adipose tissue constitutes a major contributor to hepatic lipid overload [38]. This process is further amplified by enhanced hepatic de novo lipogenesis, primarily regulated by SREBP-1c and carbohydrate-responsive element-binding protein (ChREBP), which drives transcriptional activation of key enzymes involved in fatty acid synthesis [39,40].

Concurrently, impairments in mitochondrial β-oxidation and reduced very-low-density lipoprotein (VLDL) secretion limit the liver’s capacity to dispose of excess lipids, thereby favoring intracellular triglyceride storage [41]. While triglyceride accumulation may initially serve as a protective mechanism by sequestering potentially toxic lipid species, sustained lipid overload leads to the generation of bioactive intermediates such as diacylglycerols and ceramides [41,42]. These lipotoxic molecules disrupt cellular signaling pathways, induce endoplasmic reticulum stress, and promote hepatocellular injury, thereby initiating downstream inflammatory and fibrotic responses [42,43].

At the subcellular level, lipid droplets expand in both size and number, altering organelle architecture and impairing mitochondrial function [44,45]. Excess lipid availability increases substrate pressure on mitochondrial oxidative pathways, resulting in incomplete fatty acid oxidation and enhanced production of reactive oxygen species [46]. This metabolic inflexibility compromises energy homeostasis and sensitizes hepatocytes to oxidative damage. In parallel, altered phospholipid composition of cellular membranes affects membrane fluidity and receptor signaling, further perturbing metabolic regulation. [47].

Importantly, hepatic lipid accumulation is not merely a passive storage phenomenon but actively participates in disease progression by modulating intracellular signaling networks [48,49]. Lipid-derived metabolites activate protein kinase C isoforms and stress-responsive kinases, thereby interfering with insulin signaling and reinforcing hepatic insulin resistance [50]. Furthermore, lipid-laden hepatocytes release danger-associated molecular patterns and extracellular vesicles that promote immune cell recruitment and activation, linking steatosis directly to hepatic inflammation [51].

Collectively, excessive hepatic lipid accumulation establishes a lipotoxic microenvironment characterized by disrupted metabolic signaling, mitochondrial dysfunction, and heightened cellular stress [10,43]. These alterations provide the molecular substrate for subsequent inflammatory activation and fibrotic remodeling, positioning steatosis as a critical upstream event in the MASLD continuum rather than a benign metabolic adaptation [52].

### 2.2. Insulin Resistance

Insulin resistance constitutes a central pathogenic driver linking MASLD to systemic metabolic dysfunction and plays a pivotal role in both disease initiation and progression. At the hepatic level, impaired IRS–Akt signaling disrupts the physiological actions of insulin, leading to reduced suppression of gluconeogenesis while paradoxically preserving lipogenic pathways [7]. This phenomenon, often referred to as selective hepatic insulin resistance, results in the coexistence of increased glucose production and enhanced de novo lipogenesis, thereby promoting both hyperglycemia and progressive lipid accumulation within hepatocytes [35,36,37].

Mechanistically, intracellular lipid intermediates such as diacylglycerols and ceramides activate protein kinase C isoforms and other stress-sensitive kinases, which interfere with insulin signaling cascades through inhibitory serine phosphorylation of IRS proteins [50]. These alterations impair downstream Akt activation, reducing glycogen synthesis and exacerbating hepatic glucose output [7]. Simultaneously, hyperinsulinemia continues to stimulate SREBP-1c, sustaining fatty acid synthesis despite systemic insulin resistance [17,18,19,39,40]. This dissociation between metabolic pathways creates a maladaptive state in which lipid storage is favored over glucose homeostasis.

Peripheral insulin resistance further amplifies hepatic lipid burden by increasing adipose tissue lipolysis and elevating circulating free fatty acids, which are subsequently taken up by the liver [53]. In parallel, skeletal muscle insulin resistance limits glucose disposal, contributing to chronic hyperglycemia and compensatory hyperinsulinemia [54]. Together, these extrahepatic disturbances reinforce hepatic metabolic stress and accelerate steatosis. [55,56]. Moreover, altered adipokine secretion, characterized by reduced adiponectin and increased leptin levels, exacerbates insulin resistance while promoting inflammatory and fibrogenic signaling within the liver [57].

Beyond its metabolic consequences, insulin resistance also directly contributes to hepatic inflammation and cellular injury [58]. Impaired insulin signaling enhances oxidative stress, disrupts mitochondrial function, and sensitizes hepatocytes to lipotoxic damage [10,42,43]. These effects facilitate activation of inflammatory pathways, including NF-κB and c-Jun N-terminal kinase (JNK), further aggravating insulin resistance and establishing a vicious cycle that perpetuates disease progression [9,10,11]. Collectively, insulin resistance acts not merely as a metabolic abnormality but as a key integrative mechanism linking lipid dysregulation, inflammation, and oxidative stress in NAFLD, underscoring its central role in driving the transition from simple steatosis to more advanced stages of liver disease [59].

### 2.3. Inflammation

Chronic low-grade inflammation represents a central mechanism driving the transition from simple steatosis to MASLD and plays a decisive role in amplifying hepatic injury [60]. Excessive lipid accumulation within hepatocytes triggers innate immune activation through the release of damage-associated molecular patterns, lipid metabolites, and extracellular vesicles, which stimulate Kupffer cells and recruit circulating monocytes to the liver [61]. These immune cells adopt a pro-inflammatory phenotype and secrete cytokines such as TNF-α, interleukin-1β, and IL-6, thereby establishing a sustained inflammatory milieu [61,62].

At the molecular level, these mediators activate key signaling pathways including NF-κB and c-JNK, which promote transcription of inflammatory genes and exacerbate insulin resistance through inhibitory phosphorylation of insulin receptor substrate proteins [9,10,11]. This inflammatory signaling not only impairs metabolic regulation but also induces hepatocyte apoptosis and ballooning, hallmark features of NASH [63]. In parallel, inflammasome activation, particularly through the NLRP3 complex, further amplifies cytokine production and contributes to hepatocellular injury [64].

Adipose tissue dysfunction also plays an important role in hepatic inflammation. Altered adipokine secretion, characterized by reduced adiponectin and elevated leptin levels, shifts the systemic environment toward a pro-inflammatory state [65]. While adiponectin normally exerts anti-inflammatory and insulin-sensitizing effects, its decline removes a critical protective signal, whereas increased leptin promotes immune cell activation and fibrogenic responses [66,67]. Together, hepatic and extrahepatic inflammatory inputs create a feed-forward loop that perpetuates liver damage and accelerates disease progression [68].

Beyond immune cell activation, inflammatory signaling directly influences hepatic stellate cells and endothelial cells, facilitating vascular dysfunction and fibrotic remodeling [69]. Thus, inflammation in MASLD is not merely a consequence of lipid accumulation but acts as an active driver of metabolic deterioration and structural liver injury [70].

### 2.4. Oxidative Stress and Mitochondrial Dysfunction

Oxidative stress constitutes a pivotal link between metabolic overload and inflammatory injury in MASLD [49]. Excessive fatty acid flux places substantial demands on mitochondrial oxidative capacity, leading to incomplete β-oxidation and increased generation of reactive oxygen species [71]. Additional sources of oxidative stress include peroxisomal fatty acid oxidation and cytochrome P450 enzymes, further contributing to redox imbalance within hepatocytes [72].

Mitochondrial dysfunction emerges as both a cause and consequence of oxidative stress [8,12]. Structural alterations in mitochondrial morphology, reduced respiratory chain efficiency, and impaired ATP production compromise hepatic energy homeostasis and metabolic flexibility [73]. Accumulation of reactive oxygen species induces lipid peroxidation, DNA damage, and protein modification, thereby promoting cellular dysfunction and sensitizing hepatocytes to apoptotic and necrotic pathways [74,75].

Adaptive antioxidant responses, mediated in part by Nrf2, attempt to counteract oxidative injury through upregulation of detoxifying enzymes such as superoxide dismutase, catalase, and glutathione peroxidase [11]. However, under chronic metabolic stress these compensatory mechanisms become insufficient, allowing oxidative damage to accumulate and perpetuate inflammatory signaling [49,71].

Importantly, mitochondrial dysfunction also disrupts metabolic signaling networks, impairing fatty acid oxidation and reinforcing lipid accumulation [8,12]. Altered mitochondrial dynamics and reduced biogenesis further exacerbate hepatic vulnerability, establishing a vicious cycle in which oxidative stress, mitochondrial impairment, and metabolic dysregulation mutually reinforce one another [8,12]. This convergence of redox imbalance and energetic failure plays a critical role in driving MASLD toward more advanced pathological stages [49,71].

### 2.5. Progression to NASH and Fibrosis

The progression from simple steatosis to NASH marks a critical turning point in NAFLD and is characterized by hepatocellular ballooning, persistent inflammation, and initiation of fibrotic remodeling [2,29,52]. At this stage, ongoing lipotoxicity, oxidative stress, and inflammatory signaling converge to promote widespread hepatocyte injury and death, stimulating wound-healing responses that ultimately become maladaptive [9,10,43].

Central to fibrogenesis is the activation of hepatic stellate cells, which transition from a quiescent vitamin A–storing phenotype to proliferative, collagen-producing myofibroblasts [76]. This process is driven by pro-fibrotic mediators such as transforming growth factor-beta, platelet-derived growth factor, and connective tissue growth factor [77]. Activated stellate cells deposit extracellular matrix components, including collagen types I and III, leading to progressive distortion of liver architecture [77,78].

In parallel, capillarization of hepatic sinusoids and endothelial dysfunction impair oxygen and nutrient delivery, further aggravating hepatocellular stress [79]. Persistent fibrotic remodeling disrupts normal tissue organization and culminates in cirrhosis, a state associated with portal hypertension, hepatic insufficiency, and markedly increased risk of hepatocellular carcinoma [80].

Notably, fibrosis severity rather than steatosis alone is the strongest predictor of long-term clinical outcomes in MASLD [81]. This underscores the importance of early molecular events that initiate stellate cell activation and matrix deposition [82]. Once established, fibrotic pathways become increasingly self-sustaining, highlighting the need for interventions capable of interrupting disease progression before irreversible structural damage occurs [83].

Collectively, the transition to NASH and fibrosis reflects the culmination of metabolic, inflammatory, and oxidative insults acting on the liver [84]. These interconnected processes define the advanced stages of MASLD and provide critical targets for therapeutic strategies aimed at halting or reversing disease progression [2,27,29,33].

## 3. Exercise as a Molecular Modulator of MASLD

**Figure 2:**
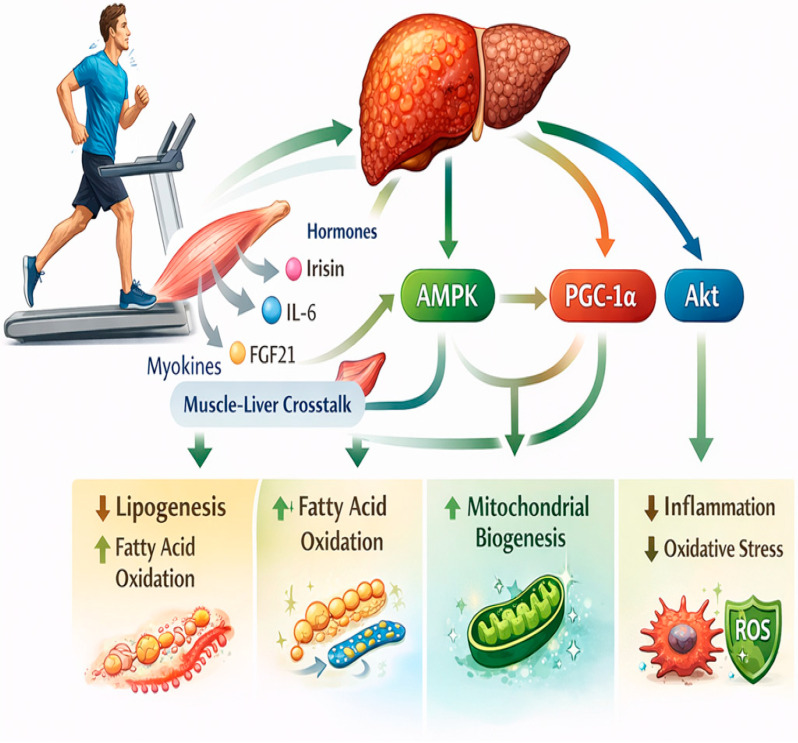
Exercise as a Molecular Modulator of MASLD. Source: own elaboration. IL-6: Interleukin-6, FGF21: Fibroblast growth factor 21, AMPK: AMP-activated Protein Kinase, PGC-1α: Peroxisome Proliferator-Activated Receptor Gamma Coactivator 1-alpha, Akt: Protein Kinase B, ROS: Reactive Oxygen Species.

Regular exercise activates central signaling pathways, including AMPK, PGC-1α, Akt, and Nrf2, resulting in enhanced mitochondrial function, increased fatty acid oxidation, reduced lipogenesis, and attenuation of inflammatory and oxidative stress. Additionally, skeletal muscle functions as an endocrine organ, releasing myokines such as irisin, IL-6, and FGF21, which communicate with the liver to further support metabolic homeostasis (Figure 2).

### 3.1. Regulation of Hepatic Glucose and Lipid Metabolism

Physical exercise has emerged as a powerful non-pharmacological intervention capable of targeting multiple pathogenic pathways involved in MASLD, acting not merely as a means of increasing energy expenditure but as a potent molecular stimulus that reprograms hepatic metabolism at systemic and cellular levels [2,14,24]. Increasing evidence indicates that exercise induces coordinated adaptations across metabolic networks, thereby restoring hepatic homeostasis and attenuating disease progression [85,86].

One of the primary molecular effects of exercise in MASLD is the regulation of hepatic glucose and lipid metabolism [87,88]. Contractile activity increases cellular energy demand, leading to activation of AMPK, a master energy sensor that orchestrates metabolic flexibility in response to energetic stress. AMPK activation suppresses SREBP-1c-mediated lipogenesis while simultaneously enhancing fatty acid oxidation through phosphorylation and inhibition of acetyl-CoA carboxylase [89,90]. This inhibition lowers malonyl-CoA concentrations, thereby relieving suppression of CPT-1 and facilitating mitochondrial fatty acid entry [91].

Concurrently, exercise improves insulin receptor substrate–Akt signaling, restoring insulin-mediated suppression of gluconeogenesis and promoting glycogen synthesis [92]. These adaptations counteract selective hepatic insulin resistance, shifting metabolic flux away from lipid storage toward substrate utilization [93]. Exercise also downregulates key lipogenic enzymes while enhancing pathways involved in lipid export and oxidation, contributing to a more favorable intrahepatic lipid profile [87,88,93]. Through these integrated mechanisms, exercise directly addresses the core metabolic disturbances underlying MASLD by re-establishing balance between lipid input, synthesis, oxidation, and disposal [88].

### 3.2. Mitochondrial Adaptations Induced by Exercise

Exercise-induced improvements in mitochondrial function represent a critical mechanism underlying its hepatoprotective effects [94]. Regular physical activity stimulates PGC-1α, a central regulator of mitochondrial biogenesis and oxidative metabolism [95]. Upregulation of PGC-1α promotes coordinated transcription of nuclear and mitochondrial genes involved in respiratory chain assembly, fatty acid oxidation, and ATP synthesis, resulting in increased mitochondrial content and enhanced bioenergetic efficiency [94,95,96].

Beyond increasing mitochondrial number, exercise favorably modulates mitochondrial quality through effects on dynamics, mitophagy, and respiratory efficiency [94]. Improved fusion–fission balance supports maintenance of functional mitochondrial networks, while enhanced turnover of damaged organelles preserves metabolic competence [94]. These adaptations restore metabolic flexibility, reduce incomplete fatty acid oxidation, and limit reactive oxygen species production [94,97].

By improving both mitochondrial quantity and quality, exercise directly addresses a central vulnerability in MASLD pathogenesis—namely impaired oxidative capacity and energetic failure—thereby reducing susceptibility to lipotoxic injury and oxidative stress [91,94].

### 3.3. Anti-Inflammatory and Antioxidant Effects

In parallel with metabolic remodeling, exercise exerts profound anti-inflammatory and antioxidant effects that counteract key drivers of MASLD progression [98]. Physical activity attenuates activation of NF-κB and c-Jun N-terminal kinase signaling pathways, resulting in reduced hepatic expression of pro-inflammatory cytokines such as tumor necrosis factor-alpha and interleukin-1β [99,100,101]. This attenuation of inflammatory signaling alleviates hepatocyte stress, limits immune cell recruitment, and improves insulin sensitivity [98].

Exercise simultaneously enhances endogenous antioxidant defenses through activation of Nrf2 [102]. Nrf2-mediated transcriptional programs upregulate detoxifying and antioxidant enzymes, including superoxide dismutase, catalase, and glutathione peroxidase, thereby reducing oxidative burden and protecting hepatocytes from lipid peroxidation and DNA damage [103].

Through coordinated suppression of inflammatory cascades and reinforcement of antioxidant capacity, exercise interrupts the feed-forward loop linking oxidative stress, inflammation, and metabolic dysfunction in MASLD [104].

### 3.4. Muscle–Liver Endocrine Communication

An increasingly recognized dimension of exercise-mediated hepatic protection is the endocrine communication between skeletal muscle and liver [105]. Contracting skeletal muscle functions as a secretory organ, releasing a diverse repertoire of myokines and signaling molecules that exert systemic metabolic effects [106]. Irisin has been implicated in enhancing fatty acid oxidation and improving insulin sensitivity, while exercise-induced IL-6 acts in a context-dependent manner, promoting lipid mobilization and anti-inflammatory signaling when released transiently from muscle [107].

FGF21, whose expression is influenced by physical activity, further contributes to hepatic lipid oxidation, mitochondrial function, and metabolic adaptation [24]. In addition to classical protein mediators, exercise modulates circulating microRNAs that regulate post-transcriptional gene expression in pathways related to lipid metabolism, inflammation, and mitochondrial biogenesis [85].

Through this muscle–liver axis, exercise synchronizes metabolic activity between peripheral tissues and the liver, reinforcing systemic insulin sensitivity and restoring energy homeostasis [108]. This inter-organ crosstalk highlights exercise as an integrated endocrine stimulus capable of orchestrating whole-body adaptations that converge on hepatic health [109].

## 4. Influence of Exercise Modalities on Molecular Pathways in MASLD

**Figure 3:**
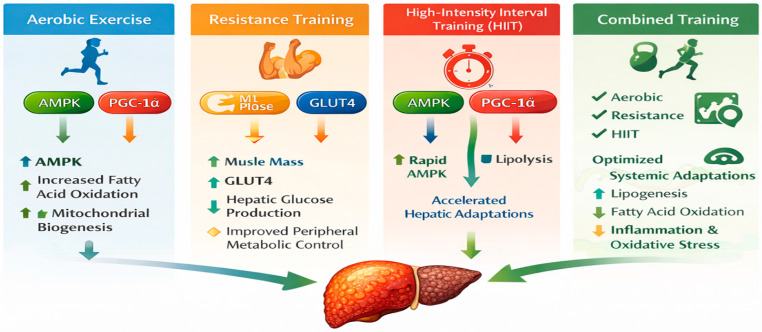
Influence of Exercise Modalities in MASLD. Source: own elaboration. AMPK: AMP-activated Protein Kinase, PGC-1α: Peroxisome Proliferator-Activated Receptor Gamma Coactivator 1-alpha, ML: Myeloid Lineage, GLUT 4: Glucose Transporter Type 4, HIIT: High-intensity interval training.

Exercise acts as a systemic stimulus, activating key signaling pathways such as AMPK, PGC-1α, Akt, and Nrf2, which together enhance mitochondrial biogenesis and fatty acid oxidation, as well as restore insulin signaling and reduce inflammatory and oxidative stress. In parallel, skeletal muscle contraction releases myokines such as irisin, IL-6, and FGF21, which mediate endocrine and paracrine communication with the liver, leading to improved metabolic flexibility and hepatoprotective effects (Figure 3).

### 4.1. Aerobic Exercise and Oxidative Metabolic Remodeling

Growing evidence suggests that distinct exercise modalities elicit partially overlapping yet mechanistically specific molecular adaptations relevant to MASLD [13,110]. Aerobic training predominantly enhances oxidative metabolism through sustained activation of AMPK and upregulation of PGC-1α, leading to increased mitochondrial biogenesis and fatty acid oxidation capacity [17,18,19,95]. Prolonged rhythmic muscle contractions promote systemic lipid mobilization and facilitate hepatic uptake and utilization of fatty acids [90,91]. These adaptations suppress hepatic de novo lipogenesis via downregulation of SREBP-1c while simultaneously increasing CPT-1 activity, thereby favoring lipid clearance from the liver [91].

Aerobic training also improves endothelial function and hepatic microcirculation, indirectly supporting oxygen delivery and mitochondrial efficiency [14,90,91,110,111]. Collectively, these mechanisms contribute to substantial reductions in intrahepatic triglyceride content and improvements in metabolic flexibility [87,88,93]. Through coordinated regulation of lipid synthesis and oxidation, aerobic exercise directly targets the metabolic core of MASLD [112].

### 4.2. Resistance Training and Peripheral Metabolic Control

Resistance training, traditionally associated with musculoskeletal adaptations, has emerged as a potent metabolic stimulus capable of improving hepatic outcomes independently of weight loss [17,18,19]. By increasing skeletal muscle mass, resistance exercise enhances whole-body glucose disposal and basal metabolic rate, thereby reducing substrate oversupply to the liver [113]. At the molecular level, resistance training activates insulin signaling pathways and promotes Glucose Transporter Type 4 (GLUT4) translocation in muscle, leading to improved systemic glycemic control [114,115].

These peripheral adaptations translate into reduced hepatic gluconeogenic drive and diminished lipid accumulation [116]. Moreover, resistance exercise stimulates secretion of myokines that modulate inflammatory tone and lipid metabolism, highlighting its endocrine contribution to liver health [105]. This modality therefore plays a critical role in addressing systemic insulin resistance and metabolic inflexibility associated with MASLD [117,118].

### 4.3. High-Intensity Interval Training and Rapid Metabolic Adaptations

High-intensity interval training (HIIT) combines brief bouts of intense effort with recovery periods, producing robust metabolic perturbations within a short time frame [119]. HIIT induces rapid AMPK activation and strong PGC-1α signaling, promoting mitochondrial biogenesis and oxidative capacity comparable to, or in some cases exceeding, that observed with traditional endurance training in healthy populations as in MASLD [17,18,19,120,121].

The intermittent high-intensity nature of HIIT enhances catecholamine-mediated lipolysis and increases fatty acid flux to oxidative tissues, facilitating hepatic lipid clearance [122]. Additionally, HIIT has been shown to improve insulin sensitivity and reduce inflammatory markers despite lower total exercise volume, making it an attractive time-efficient strategy for individuals with metabolic disease [123].

### 4.4. Combined Training Approaches and Integrated Molecular Benefits

Although each modality engages distinct primary pathways, all forms of exercise converge on shared molecular targets relevant to MASLD, including suppression of inflammatory signaling, activation of antioxidant defenses via Nrf2, and improvement of mitochondrial quality [104]. Importantly, exercise-induced reductions in hepatic fat can occur in the absence of significant weight loss, underscoring the direct molecular effects of physical activity on liver tissue [17,18,19].

Emerging evidence suggests that combined training approaches may provide additive or synergistic benefits by simultaneously targeting oxidative capacity and muscle mass [124]. Integrative programs incorporating aerobic and resistance components appear particularly effective in optimizing metabolic control, reducing inflammation, and enhancing functional capacity [125]. Such multimodal strategies may therefore represent an optimal framework for personalized exercise interventions in MASLD [6,33,39].

Collectively, these findings highlight that while aerobic, resistance, and HIIT paradigms differ in their mechanistic emphasis, each modality exerts meaningful effects on the molecular networks driving MASLD [13,20,117,118,120,121,126]. This convergence supports the concept that exercise prescription should be flexible and individualized, prioritizing adherence while leveraging modality-specific adaptations to maximize therapeutic impact [13,20].

## 5. Clinical and Translational Implications of Exercise in MASLD

The expanding understanding of exercise-induced molecular adaptations provides a compelling framework for translating mechanistic insights into clinical strategies for MASLD management [127]. By simultaneously targeting hepatic metabolism, mitochondrial function, inflammatory signaling, and inter-organ communication, exercise addresses the multifactorial nature of MASLD more comprehensively than most pharmacological approaches [3,4,6]. These pleiotropic effects support the integration of structured physical activity as a cornerstone intervention across the entire disease spectrum, from early steatosis to advanced fibrosis [127,128].

Importantly, exercise-mediated reductions in hepatic fat content and improvements in insulin sensitivity frequently occur independently of significant weight loss, emphasizing that metabolic remodeling rather than body mass reduction per se drives hepatic benefits [129,130]. This observation has substantial clinical relevance, as it broadens therapeutic opportunities for patients who struggle to achieve or maintain meaningful weight loss [130]. Moreover, improvements in cardiorespiratory fitness and muscle strength confer additional protective effects against cardiovascular morbidity, a leading cause of mortality in individuals with MASLD [131,132].

From a translational perspective, emerging biomarkers reflecting exercise responsiveness may facilitate individualized intervention strategies [6]. Circulating levels of FGF21, irisin, inflammatory cytokines, and oxidative stress markers, together with profiles of exercise-regulated microRNAs, hold promise as indicators of hepatic metabolic adaptation [24,85]. Advanced imaging modalities and non-invasive fibrosis scores further enable monitoring of structural and functional liver changes in response to training [133]. Integration of these biomarkers into clinical practice may allow stratification of patients based on molecular phenotype and predicted responsiveness to specific exercise modalities [17,18,19].

The heterogeneity of MASLD underscores the need for personalized exercise prescriptions [134]. Patients differ markedly in metabolic status, fibrosis stage, physical capacity, and comorbidity burden, necessitating tailored programs that balance efficacy with adherence and safety [134,135]. Aerobic exercise may be prioritized in individuals with pronounced steatosis and mitochondrial dysfunction, whereas resistance training may offer particular benefit in sarcopenic or insulin-resistant phenotypes [14,87,88,90,91,93,110,111,112].

HIIT provides a time-efficient alternative for selected patients, while combined training approaches may maximize systemic and hepatic adaptations [120,121,122]. Such precision-based frameworks align with contemporary concepts of personalized medicine and may enhance long-term treatment success [123].

Exercise also exhibits substantial synergistic potential when combined with dietary interventions and emerging pharmacotherapies [136]. Nutritional strategies that reduce hepatic substrate overload may amplify exercise-induced metabolic remodeling, while pharmacological agents targeting fibrosis or inflammation could complement the upstream benefits of physical activity [35,125,136]. Multimodal approaches therefore represent a promising direction for comprehensive MASLD management, integrating lifestyle and medical therapies within a unified mechanistic framework [6].

Despite robust evidence supporting exercise efficacy, implementation remains a major challenge [137]. Barriers include limited access to supervised programs, variable patient motivation, and insufficient integration of exercise specialists into hepatology care pathways [138]. Addressing these gaps will require interdisciplinary collaboration and development of scalable models that embed structured physical activity within routine clinical practice [138,139]. Digital health platforms and remote monitoring technologies may further facilitate adherence and enable real-time adjustment of exercise prescriptions [140].

**Figure 4:**
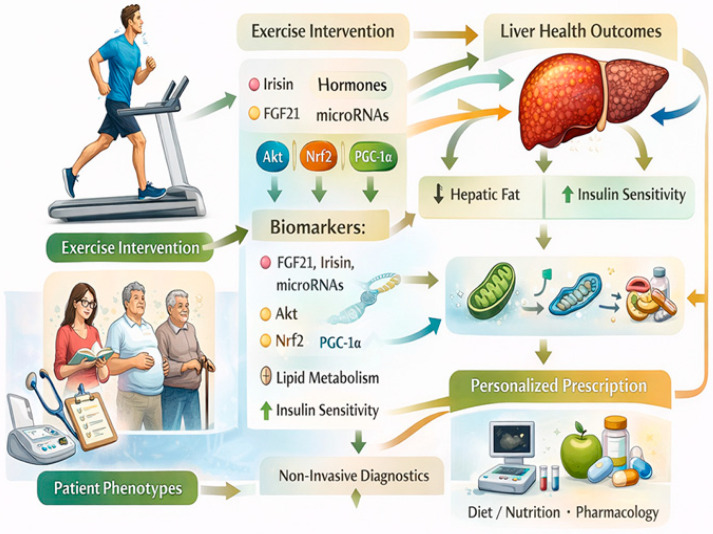
Clinical and Translational Implications. Source: own elaboration. FGF21: Fibroblast growth factor 21, Akt: Protein Kinase B, Nrf2: Nuclear factor erythroid 2-related factor 2, PGC-1α: Peroxisome Proliferator-Activated Receptor Gamma Coactivator 1-alpha.

Collectively, current evidence positions exercise not merely as an adjunct lifestyle recommendation but as a biologically active therapy with disease-modifying potential in MASLD [130,137]. By leveraging its capacity to simultaneously modulate metabolic, inflammatory, oxidative, and endocrine pathways, exercise offers a uniquely integrative approach to liver health [130,137]. Future research should focus on refining modality-specific prescriptions, identifying molecular predictors of response, and establishing standardized clinical protocols to fully realize the translational potential of exercise-based interventions in MASLD (Figure 4).

## 6. Conclusions

Exercise represents a potent, disease-modifying intervention in MASLD, capable of simultaneously improving hepatic metabolism, mitochondrial function, insulin sensitivity, and inflammatory balance. Aerobic, resistance, and HIIT modalities elicit complementary molecular adaptations, often independent of significant weight loss, while combined or individualized programs can maximize systemic and hepatic benefits. By integrating mechanistic insights with personalized approaches, exercise offers a biologically active strategy to address the multifactorial pathophysiology of MASLD and improve long-term metabolic and liver health.

## Data Availability

No new data were created or analyzed in this study. Data sharing is not applicable to this article.
